# Feasibility, safety and outcomes of contrast-free cardiac resynchronisation therapy device implantation: a retrospective cohort comparison

**DOI:** 10.1007/s10840-026-02283-0

**Published:** 2026-03-24

**Authors:** L. Friedrich, M. Walgenbach, M. O`Connor, A. Fetcu, F. Geißenberger, T. Trenkwalder, G. Krause, C. Mronga, H. Schunkert, C. Kolb, A. Chouchane, Carsten Lennerz

**Affiliations:** 1 Department of Cardiovascular Diseases, German Heart Center, School of Medicine and Health, TUM University Hospital, Technical University of Munich, Munich, Germany; 2 Department of Electrophysiology, Te Toka Tumai Auckland City Hospital, Auckland, New Zealand; 3 Department of Cardiology, University of Oldenburg, Klinikum Oldenburg, Oldenburg, Germany; 4 DZHK (German Centre for Cardiovascular Research), partner site Munich Heart Alliance, Munich, Germany; 5 Klinik für Herz- und Kreislauferkrankungen, TUM Universitätsklinik Deutsches Herzzentrum, Lazarettstr. 36, 80636 München, Germany

**Keywords:** Cardiac implantable electronic devices, Cardiac resynchronization therapy, CRT, Left ventricular lead implantation, Coronary sinus, Contrast-free implantation, Fluoroscopy

## Abstract

**Background:**

Left ventricular lead (LVL) implantation can be challenging in Cardiac Resynchronization Therapy (CRT). Therefore, contrast is commonly used to visualize the coronary venous system. However, contrast use is correlated with substantial risks such as contrast-induced nephropathy and anaphylactic reaction. Case studies suggest feasibility of LVL implantation without coronary sinus (CS) venography.

**Purpose:**

We aimed to compare the procedure characteristics, outcome, and safety of contrast-free and contrast-aided LVL implantation.

**Methods:**

346 LVL implantations performed between 2017 and 2019 were analysed. 167 were attempted without contrast (Intervention Group: IG), and 179 were fully performed with contrast (Control Group: CG). The data was collected and analysed retrospectively from a single centre. These two groups were compared in an Intention-To-Treat (ITT) analysis. An As-Treated (AT) analysis was performed to compare procedures fully performed without contrast (NCG), crossover procedures (COG) and the control group (CG). The intervention duration, fluoroscopy duration, and radiation dose were evaluated as procedure characteristics. The primary outcome was procedural success. The secondary outcomes were LVL position, LVL threshold value, QRS duration and LVEF evolution. Perioperative complications and lead revisions were surrogates for the intervention’s safety.

**Results:**

The overall LVL implantation success rate was 97.7%. Of the contrast-free procedures 62.9% were successful, the remainder required crossover to contrast use compared to 96.6% with contrast use. The ITT and AT analyses yielded similar results. Contrast-free interventions were associated with shorter procedure (100.8 ± 41.0 vs. 131.1 ± 50.0 min, *p* < 0.01), less fluoroscopy (15.7 ± 11.2 vs. 26.0 ± 17.5 min, *p* < 0.01) and smaller radiation doses, (475.3 ± 422.7 vs. 897.3 ± 779.1 cGy.cm2, *p* < 0.01). The intraoperative LVL threshold values were equivalent (0.9 ± 0.6 in the NCG vs. 1.0 ± 0.6 V in the CG, TOST-*p* < 0.01), and contrast use did not significantly influence the follow-up LVL threshold (1.1 ± 0.5 vs. 1.2 ± 0.9 V (*p* = 0.62), QRS shortening (10.5 ± 28.3 vs. −13.2 ± 26.3ms, *p* = 0.16) or LVEF increase − (2.3 ± 8% vs. 4.1 ± 8.4%, *p* = 0.40). More CS dissections were observed during the contrast-aided procedures (5% in the CG versus 3.2% in the COG versus 0% in the NCG, *p* = 0.03). However, the perioperative complication rates (pericardial effusion, ICU admission, death, mechanically induced arrhythmia, and pneumothorax) did not significantly differ (*p* > 0.05 for each). In contrast-free LVL implantation failure cases, intraoperatively crossing over from the NCG to CG did not result in longer procedures (131.8 ± 46.8 in the COG vs. 131.1 ± 50.0 min in the CG, *p* = 0.90). Although more adverse events were observed in the CG, the difference was not statistically significant (6.5% in the COG vs. 12.3% in the CG, *p* = 0.20).

**Conclusion:**

Contrast-free LVL implantation was associated with shorter intervention durations, less fluoroscopy and lower radiation doses. Fewer intraoperative complications were observed without contrast, and the postoperative complication rate was similar to contrast-aided procedures. This technique was successful in almost 2/3 of the cases. Where a crossover to contrast use was required, the initial contrast-free approach did not result in a statistically significant increase in clinically relevant adverse events compared with fully contrast-aided procedures. It is therefore reasonable to consider an initial contrast-free approach for CRT implantation.

**Graphical Abstract:**

Central Illustration: Feasibility, procedural characteristics, and lead positioning of contrast-free LV lead implantation.

**Figure:**
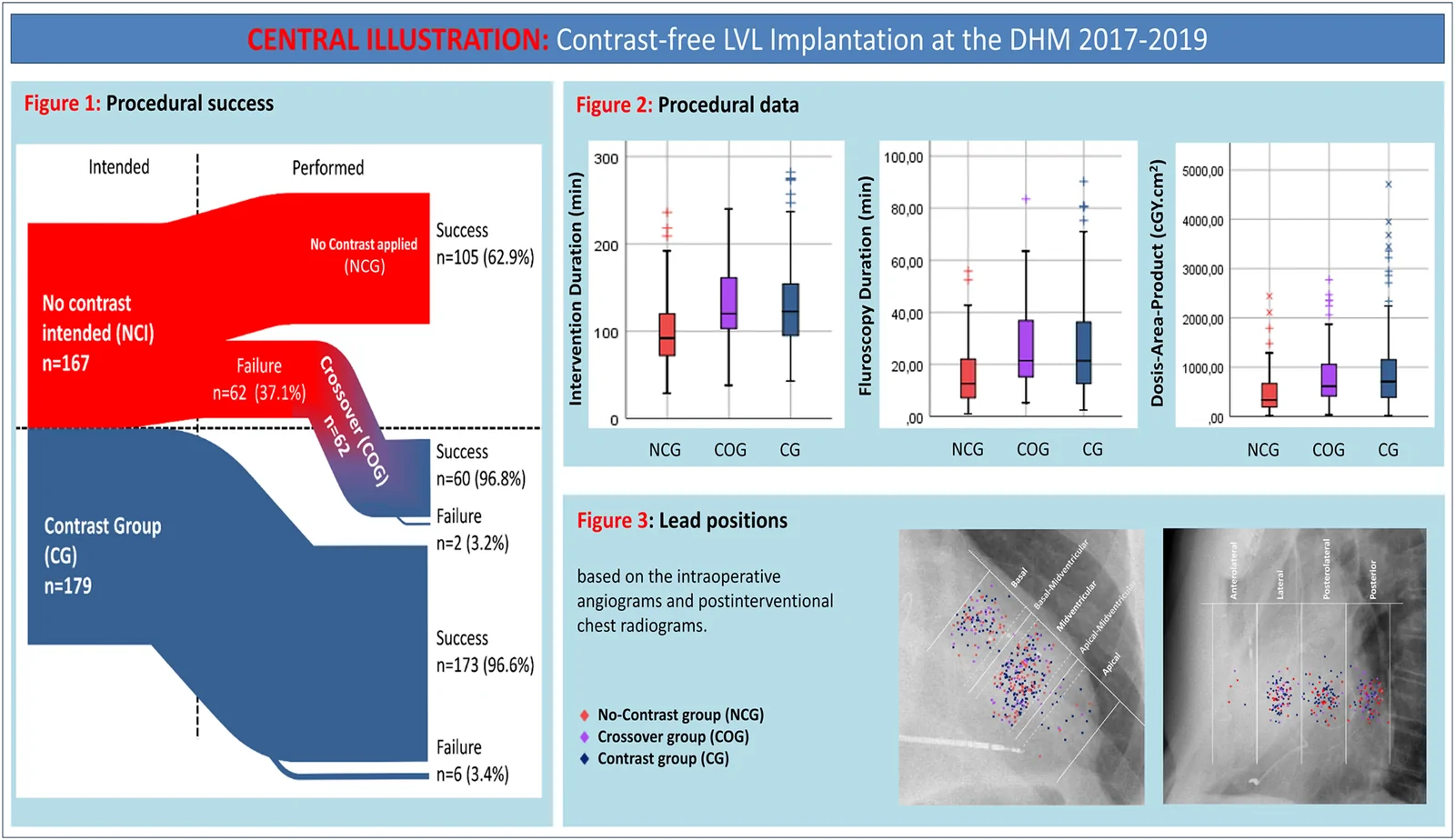

## Introduction

Cardiac resynchronisation therapy (CRT) is a well-established therapy for severe cardiomyopathies with left bundle branch block (LBBB) and pacing-induced cardiomyopathy resulting in improved mortality and hospitalisation rates 1, 2. This technique involves placing a pacing lead in the right ventricle (RV) and a second ventricular lead on the epicardial surface of the left ventricle (LV) via the coronary sinus (CS) and its branches. The LV lateral wall can then be stimulated earlier than intrinsic LBBB conduction or RV pacing would otherwise result in, thus providing more synchronous LV stimulation and contraction.

**Fig. 1:**
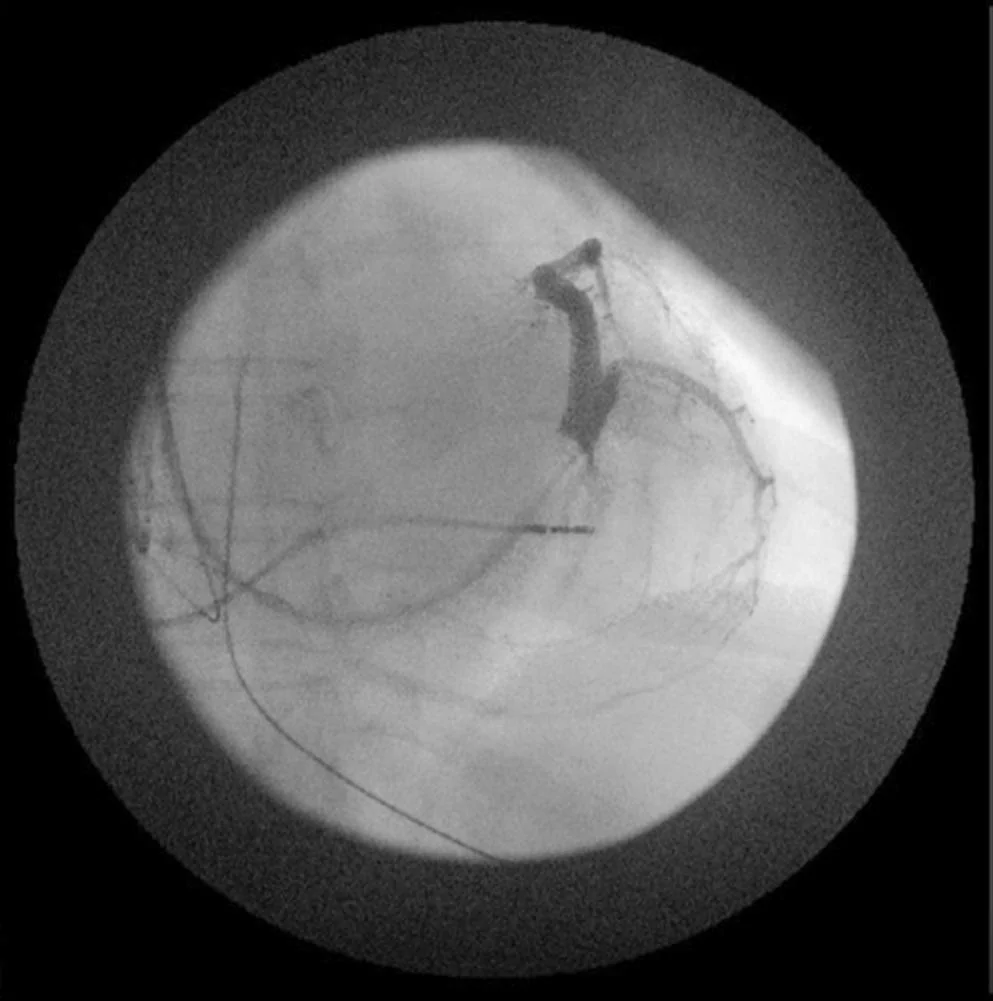
Venography of the coronary sinus. Balloon occlusion venography of the CS during CRT implantation

The coronary venous anatomy, upon which LV lead placement is dependant, is highly variable. Currently, the gold standard for delineating this anatomy is retrograde CS venography using iodinated contrast medium (CM), performed either with an occlusive balloon (occlusive venography) or without a ballon (direct venography) (Fig. 1). The use of CM is associated with the development of acute kidney impairment (AKI) in the form of contrast-induced nephropathy. This is of particular concern in the CRT population as LV impairment and chronic renal impairment often co-exist placing these patients at high risk of post-CRT AKI; this risk may be up to 15% and is higher in those with larger CM doses 3–5. Post-CRT AKI is also associated with a significantly higher mortality^4^. Furthermore, de-novo contrast allergies that may result in severe allergic reactions are idiosyncratic and unpredictable. Patients with known contrast allergies must be pre-treated with steroids and antihistamines to reduce, but not exclude, the risk of a reaction complicating pre-operative management.

Techniques to aid LV lead placement in CRT implantation have been developed, such as the use of echo, CT, MRI to identify the coronary venous anatomy, region of latest activation and scar distribution 6–10. Contrast free CRT implantation has also been shown to be feasible using electroanatomical mapping in case reports 11, 12, but the availability of electroanatomical mapping systems is not common in most device labs and the use of such mapping significantly increases procedure time. The feasibility of completely contrast-free CRT device implantation has been reported 13. However, the ability to routinely implant CRT devices without the use of contrast medium, the procedural details and the outcomes of these interventions has not been extensively evaluated.

We outline our technique of contrast-free LV lead placement and present our data of a matched cohort of patients undergoing CRT with or without the use of contrast medium.

## Methods

This study included all patients that underwent LVL implantation between 01/01/2017 and 12/31/2019 at the German Heart Centre in Munich (DHM). The data was collected retrospectively. The inclusion criteria were indication for de-novo CRT implantation, indication for device upgrade to CRT and indication for LVL revision. The indications for CRT implantation were based on the contemporary European Society of Cardiology (ESC) guideline^14^. Patients younger than 18 were excluded. We identified the cases from the device implantation databank of the DHM.

The patients’ characteristics and past medical history were retrieved from the electronic patient files. Serum creatinine levels were obtained from the laboratory records, and the glomerular filtration rate (GFR) was calculated using the Modification of Diet in Renal Diseases (MDRD) formula 15. The last obtained preoperative GFR was considered as baseline. The lowest in-hospital GFR after the procedure was reported as postoperative GFR. Acute kidney injury (AKI) was defined as a ≥ 1.5-fold creatinine increase within seven days or a creatinine level increase of 0.3 mg/dL within 48 h compared to pre-procedural value 16.

The procedure characteristics, consisting of the contrast volume, procedure duration, fluoroscopy duration, dose area product, and LVL intraoperative threshold and impedance, were extracted from the device implantation databank. Missing contrast volume entries were retrieved from the procedure logbooks. In this observational study, the decision to use or not to use contrast medium for LVL implantation was left to the discretion of the implanting interventionalist. All procedures were performed by three experienced CIED specialists, each with more than 10 years of experience in device implantation and an annual volume of approximately 30–50 CRT implantations. CS cannulation was performed using a guiding CS sheath (Abbott CS catheter with 135° curve, Boston Scientific Acuity Hook, Biotronik Selectra Hook, or Medtronic Attain Command delivery system) in combination with an inner Amplatz Left II catheter and a 0.035″ guidewire. No electrophysiology (EP) diagnostic catheter was used for CS cannulation.

We reviewed the procedure notes and fluoroscopy videos to assess at which step of the procedure contrast was injected. Procedures where contrast was only used to establish brachial/cephalic vein access were considered contrast-free LVL implantations. Further, we assessed if contrast was directly used to visualize the coronary sinus (CS) tributaries following its cannulation or if contrast-free LVL implantation was attempted and failed before an intraoperative crossover to contrast-aided implantation was performed. This review yielded three comparison groups: contrast-free procedures (NCG), contrast-free to contrast-aided crossover procedures (Crossover group: COG), and procedures where contrast was immediately injected following CS cannulation (Contrast Group: CG).

We reviewed the fluoroscopy videos and the postoperative chest x-rays to assess the LVL positions. On the frontal projection, the lead positions were categorized into basal, basal-midventricular, midventricular, apical-midventricular and apical. On the lateral projection, the positions were identified as anterior, anterolateral, lateral, posterolateral, and posterior 17. We remeasured the QRS duration (QRS_d_) on digitalized ECGs in the Schiller SEMA V2.70.1.1 environment. QRS_d_ was defined as the duration of the widest QRS in any lead 18. The last obtained preoperative ECG was considered as baseline. The postoperative ECGs were obtained during the postoperative care period. The postoperative LVL threshold and impedance values were obtained from the device follow-up databank—values measured during the hospitalization and three months after the implantation were included in the analysis. The LVEF values were retrieved from the echocardiography databank. The last obtained preoperative LVEF was considered baseline. The LVEF follow-up values from 01/01/2017 to 12/31/2020 were extracted when available, and the first postoperative LVEF recorded at least two months after the procedure was maintained as follow-up LVEF.

Intraoperative complications (CS dissection and mechanically induced arrhythmias, as well as implantation failure, were assessed by reviewing the procedure notes and the fluoroscopy films. Mechanically induced arrhythmias were defined as ventricular arrythmias (non-sustained or sustained ventricular tachycardia or ventricular fibrillation) occurring during the LV lead positioning within the coronary sinus and attributed to mechanical stimulation of the myocardial tissue (e.g. stretch, pressure), which may activate mechanosensitive ion channels^19^, rather than to primary electrical or metabolic causes.

Perioperative complications (pericardial effusion, pneumothorax, ICU admission and death) were assessed by reviewing the postoperative test reports and hospitalization records. To assess if LVL revisions occurred since the implantation, the serial number of the LVL at every follow-up performed from the implantation date to 12/31/2020 was compared with the LVL serial number documented at the procedure. A mismatch between the LVL serial number at implantation and follow-up indicated an LVL revision.

### Statistical analysis

Due to the intraoperative crossover from contrast-free to contrast-aided implantation in some cases, we performed an Intention-To-Treat (ITT) and an As-Treated (AT) analysis. From the ITT perspective, the intervention group (IG) contains the procedures attempted without contrast and represents the union of the no-contrast implantation group (NCG) and the crossover group (COG), while the control group includes the procedures that were intended and performed with contrast (CG). In the AT analysis, the three groups (NCG, COG and CG) were separately reported and compared.

We used IBM SPSS Statistics Version 25 for statistical analysis. We reported the mean ± standard deviation for continuous variables. For discrete variables, we reported the median and interquartile range (IQR). The absolute and relative frequencies were reported for categorical data. Missing values were omitted from the analysis. The statistical tests were two-tailed, and a significance threshold for type-1-error α = 0.05 was assumed.

T-tests, analysis of variance (ANOVA) tests with Welch correction and Turkey HSD posthoc tests were performed on normally distributed data, except: (1) For the intervention duration, linear regression was performed to analyse the association between the intervention duration and the use of contrast. The analysis was adjusted for the interventionist and the patient characteristics. An interaction term between the interventionalist and the use of contrast was added to evaluate any effect modification by the interventionalist. (2) For the intraoperative LVL threshold, two one-sided t-tests (TOST) were performed to compare the NCG and the CG values. For α = 0.05, power β = 90% and a minimal sample size of 100 observations per group, we set the smallest effect size of interest (SESOI) at Cohen’s d = 0.4 20. This analysis was performed using the TOSTer package of Jamovi Version 2.3.12. (3) Repeated measure ANOVA was performed to assess the association between contrast use and the evolution of the LVL threshold, impedance, QRS duration, LVEF and GFR.

We performed Chi-squared (χ2) tests on categorical data. When the χ2 expected frequency assumption was inappropriate, Fisher-Freeman-Halton tests were used 21.

Binary logistic regression was performed to analyse the association between AKI and contrast use. The analysis was adjusted for the patient characteristics (age, sex, BMI, preoperative GFR and LVEF), the interventionalist and the preoperative creatinine level.

## Results

### Patient population

**Fig. 2:**
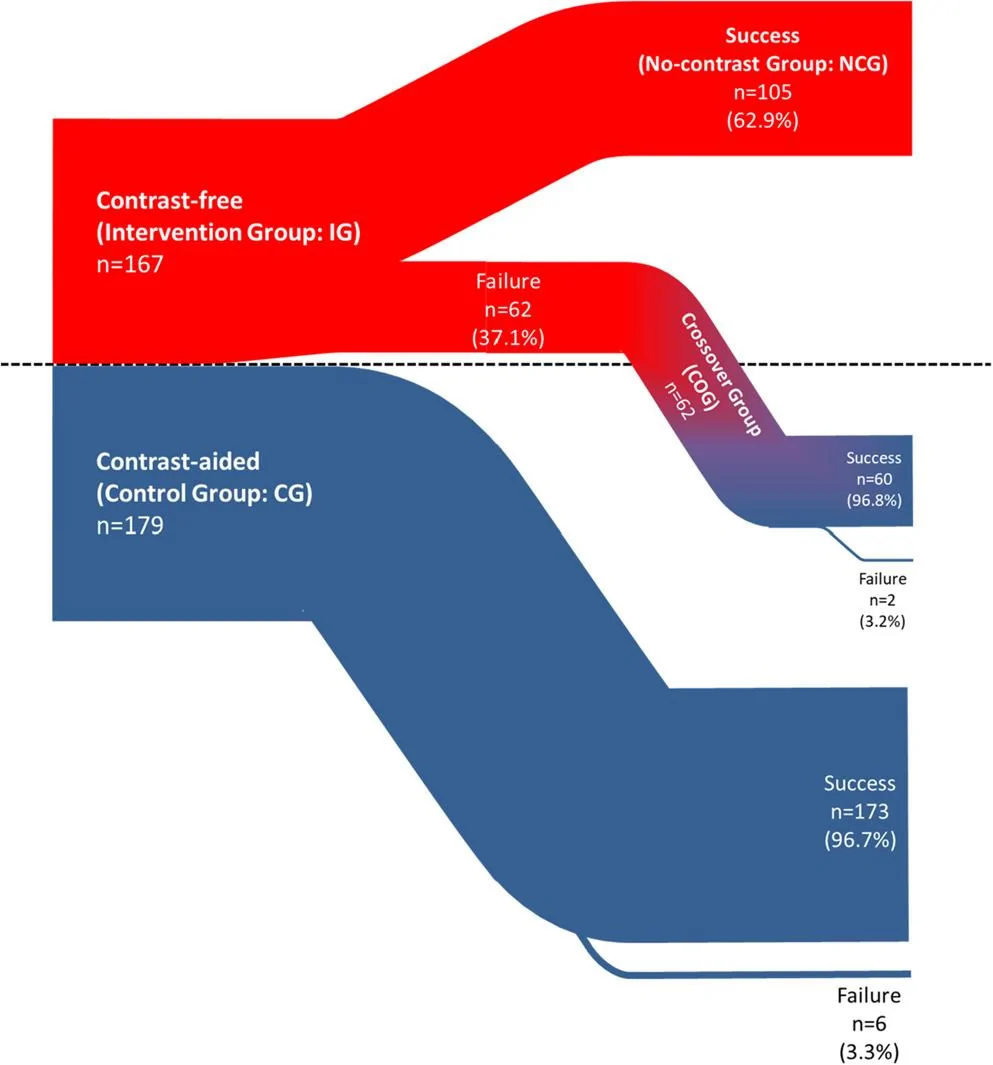
Compared groups and corresponding success rates. 346 interventions were performed. The success rate was 96.6% (173/179) in the CG and 62.9% (105/167) in the NCG group. In the crossover from contrast-free to contrast-aided LVL implantation, 96.8% (60/62) of LVL implants were successful. The total success rate was 97.7% (338/346)

We analysed 346 patients that underwent LVL implantation. The control group (CG), where contrast was injected directly upon CS cannulation, included 179 (51.7%) cases. The intervention group (IG), where contrast-free implantation was attempted, contained 167 cases (48.3%). Among these, 105 cases (62.9%) were successfully contrast-free (NCG), and 62 cases (37.1%) required a crossover from contrast-free to contrast-aided implantation (COG). The groups are detailed in Fig. 2.

The ITT analysis showed that contrast use was associated with higher BMIs (28.0 ± 6.0 kg/m2 vs. 26.2 ± 4.1 kg/m2, *p* < 0.01), lack of contrast counterindications such as hyperthyroidism (*p* = 0.03), contrast allergy (*p* = 0.02) and with device upgrade procedures (*p* = 0.03). The preoperative QRSd in the CG was longer than in the intervention group IG (157.6 ± 29.1 ms vs. 149.8 ± 27.1 ms, *p* = 0.03). The other patient characteristics did not significantly differ between the groups including age, sex, preoperative LVEF, and preoperative creatinine. The association between renal insufficiency and contrast use was not significant in the studied population (*p* = 0.20); overall, 54% of the patients had a GFR ≤ 60 mL/min/1.73m2. Contrast use was also not associated with the device type. The AT analysis showed similar results for the patient and device characteristics, as detailed in Table 1.

**Table 1:** Patient and CRT characteristics

			Intention-to-treat			As-treated			
			IG* **(** *n* ** = 167)**	CG **(** *n* ** = 179)**	*p*-value	NCG **(** *n* ** = 105)**	COG **(** *n* ** = 62)**	CG **(** *n* ** = 179)**	*p*-value
Patient characteristics									
	Age (years)		70.3 ± 14.8	68.5 ± 14.5	0.26	69.7 ± 14.8	71.2 ± 14.8	68.5 ± 14.5	0.44
	Sex (male)		126 (75.5%)	125 (69.8%)	0.19	78 (74.3%)	48 (77.4%)	125 (69.8%)	0.37
	BMI (kg/m^2^)		26.2 ± 4.1	28.1 ± 6.0	**< 0.01**	26.2 ± 4.1	26.3 ± 4.1	28.1 ± 6.0	**< 0.01**
	Preoperative Creatinine (mg/dL)		1.4 ± 0.6	1.3 ± 0.6	0.06	1.4 ± 0.6	1.3 ± 0.5	1.3 ± 0.6	0.12
	Preoperative QRS (ms)		149.8 ± 27.1	157.7 ± 29.0	**0.02**	150.7 ± 26.1	148.2 ± 28.8	157.7 ± 29.1	0.06
	Preoperative LVEF (%)		31.0 ± 7.6	30.8 ± 7.9	0.89	29.9 ± 7.8	33.0 ± 6.6	30.8 ± 7.9	0.59
	Comorbidities								
		Hyperthyroidism	16 (9.6%)	7 (3.9%)	**0.03**	12 (11.4%)	4 (6.5%)	7 (3.9%)	**< 0.01**
		Contrast Allergy	9 (5.4%)	2 (1.1%)	**0.02**	9 (8.6%)	0 (0%)	2 (1.1%)	**< 0.01**
		Renal Insufficiency (GFR ≤ 60)	96 (57.5%)	91 (50.8%)	0.2	62 (59%)	34 (54.8%)	91 (50.8%)	0.42
CRT characteristics									
	CRT-Type				0.08				0.93
		CRT-D	119 (71.2%)	130 (72.6%)		74 (70.5%)	45 (72.1%)	130 (72.6%)	
		CRT-P	48 (28.8%)	49 (27.4%)		31 (29.5%)	17 (27.9%)	49 (27.4%)	
	Procedure Type				**0.03**				**0.01**
		De-Novo	100 (59.9%)	86 (48.0%)		61 (58%)	39 (62.9%)	86 (48.0%)	
		Upgrade	67 (40.1%)	93 (51.9%)		44 (41.9%)	23 (37.1%)	93 (51.9%)	

*IG* Intervention group, *CG* Control Group = Contrast Group, *NCG* No Contrast group, *COG* Crossover Group

*IG=NCG+COG; This group consists of the procedures attempted without contrast

## Procedure characteristics

Overall, the mean procedure duration was 123 ± 32.3 min, the mean fluoroscopy duration was 22.9 ± 16.2 min, and the mean dose area product was 758.8 ± 681.4 cGy.cm2. Comparing these characteristics across the groups revealed significant differences (*p* < 0.01 for each), as detailed in Table 2.

**Table 2:** Procedure characteristics

	Intention-to-treat			As-treated			
	IG	CG	*p*-value	NCG	COG	CG	*p*-value
Contrast volume (mL)	13.7 ± 21.5	44.1 ± 35.6	**< 0.01**	1.9 ± 7.8*	33.6 ± 22.7	44.1 ± 35.6	**< 0.01**
Intervention duration (min)	112.2 ± 45.6	131.1 ± 50.0	**0.02**	100.8 ± 41.0	131.7 ± 46.7	131.1 ± 50.0	**< 0.01**
Fluoroscopy Duration (min)	19.8 ± 14.3	25.9 ± 17.5	**< 0.01**	15.7 ± 11.2	26.9 ± 16.2	25.9 ± 17.5	**< 0.01**
Dose area product (cGy.cm2)	613.9 ± 545.6	897.3 ± 779.2	**< 0.01**	475.3 ± 422.6	852.4 ± 646	897.3 ± 779.2	**< 0.01**

* When contrast was used only for visualization of the brachial/subclavian vein (not for CS visualization), the procedure was considered as a contrast-free procedure

 The ITT analysis showed that the procedures attempted without contrast were significantly shorter (procedure duration: 112.2 ± 45.6 min in the NCG versus 131.1 ± 50.0 min in the CG) and associated with less radiation exposure than the procedures intended with contrast (fluoroscopy use 19.8 ± 14.3 min in the NCG versus 25.9 + 17.5 min in the CG, and radiation dose product 613.9 ± 545.6 cGy.cm2 in the NCG versus 897.3 ± 779.2 cGy.cm2 in the CG). The AT analysis showed more pronounced differences between the contrast-free and the contrast-aided procedures regarding the procedure duration (100.8 ± 41 min vs. 131.1 ± 50.0 min), fluoroscopy use (15.7 ± 11.2 min vs. 25.9 + 17.5 min) and radiation dose (475.3 ± 422.6-cGy.cm2 vs. 897.3 ± 779.2-cGy.cm2). In case of contrast-free implantation failure, the crossover from contrast-free to contrast-aided LVL implantation yielded similar intervention durations (*p* = 0.99), fluoroscopy durations (*p* = 0.92) and dose area products (*p* = 0.89) compared to the CG, although the used contrast volume in the COG was lower (33.6 ± 22.7 mL vs. 44.1 ± 35.6mL).

Linear regression modelling of the intervention’s duration based on AT contrast use (NCG vs. COG vs. CG) and the interventionalist showed that the contrast use and the interventionalist’s experience independently affected the intervention’s duration (*p* < 0.01 for both). The interaction between the two factors was, however, not significant (*p* = 0.30). The analysis was adjusted for the patient’s characteristics and the procedure type. The ITT linear model of the intervention duration showed similar results (*p* = 0.02 for contrast use, *p* = 0.01 for the interventionalist and *p* = 0.96 for interaction). A linear regression analysis performed on the fluoroscopy duration similarly showed that contrast use (NCG vs. COG vs. CG) and the interventionalist independently influenced the fluoroscopy duration (*p* < 0.01 for both) and that their interaction was not significant (*p* = 0.71). ITT linear modelling of the fluoroscopy duration showed similar results (*p* = 0.02 for contrast use, *p* < 0.01 for the interventionalist, and *p* = 0.09 for interaction).

## Intraoperative outcome

The LVL implantation ITT success rate was 96.7% (173/179) in the CG and 62.9% (105/167) in the IG (*p* < 0.01). All the failed contrast-free implantations (62/167) were intraoperatively converted to contrast-aided procedures. The crossover yielded a 96.8% (60/62) LVL implantation success rate in the COG. Overall, 97.7% (338/346) of the interventions ended with successful LVL implantation.

**Fig. 3:**
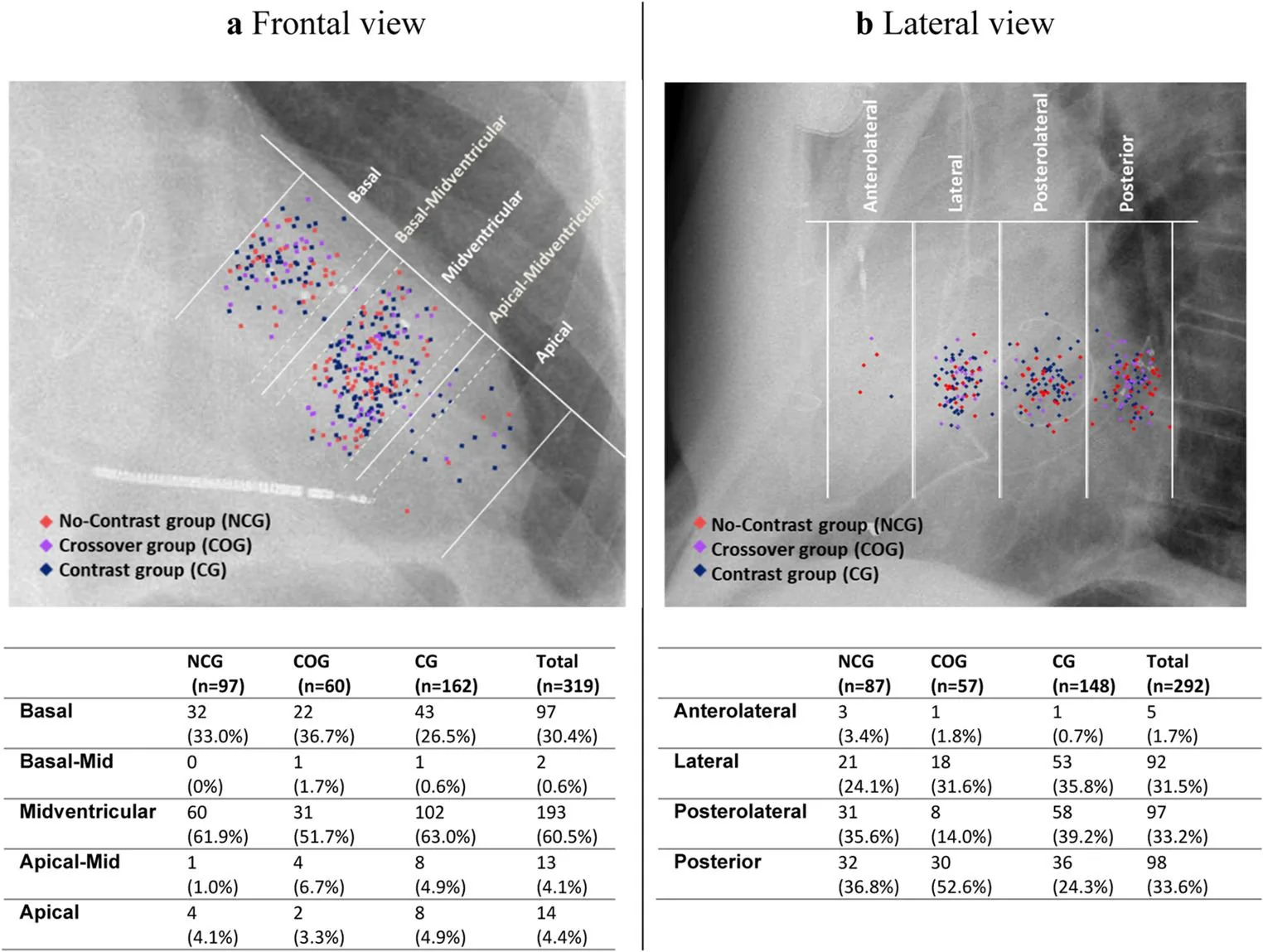
Distribution of the LVL positions on the postoperative radiographs. The LVL was successfully implanted in 338 cases. 319 frontal and 292 lateral postoperative x-ray projections could be retrieved. The LVL positions were not associated with contrast use on the frontal projection (*p*=0.27). On the lateral projection, the association was significant (*p*<0.01)

The ITT and AT analyses showed that the LVL positions were not associated with contrast use on the frontal plane (respectively *p* = 0.47 and *p* = 0.27). The association was, however, significant on the lateral plane (*p* < 0.01 for both analyses). The AT analysis showed that the most frequent LVL positions on the frontal projection were midventricular (60.5%) and basal (30.4%). On the lateral projection, the most frequent positions were posterior (33.6%), posterolateral (33.2%) and lateral (31.5%). The LVL positions on this projection were associated with contrast use (*p* < 0.01). The use of contrast led to more lateral lead positions (35.8% vs. 31.6% vs. 24.1%, (CG, COG, NCG respectively)). When performing LVL implantations without contrast, more posterior lead positions (36.8% vs. 52.6% vs. 24.3%, %, (CG, COG, NCG respectively)) and anterolateral positions (3.4% vs. 1.8% vs. 0.7% (NCG, COG, CG respectively)) were obtained. Figure 3 details the distribution of the LVL positions within the compared groups.

An equivalence test (TOST) was performed to compare the threshold values of the contrast-free implants (NCG, mean = 0.97 ± 0.6 V) and the contrast-aided implants (COG + CG, mean = 1.1 ± 0.6 V). It demonstrated equivalence (upper TOST *p* < 0.01, lower TOST *p* = 0.03). Performing an ITT equivalence test to compare the thresholds of the implants attempted without contrast to the thresholds of implants intended with contrast yielded similar results. There was no difference in intraoperative impedance values between the three studied groups (*p* = 0.89).

### Postoperative evolution

Overall, the QRS duration decreased from 154 ± 28 ms to 143 ± 24 ms(*p* < 0.01). Comparing the three groups (NCG vs. COG vs. CG) showed that contrast use did not represent a significant effect (*p* = 0.16) on the QRS shortening. The mean QRS shortening was similar across groups (−13.1 ± 26.3ms in the CG, −10.4 ± 28.3ms in the NCG and − 3.9 ± 25.6ms in the COG).

The LVL threshold was 1.0 V ± 0.6 V intraoperatively, 1.2 V ± 1.0 V during the hospitalization and 1.2 ± 0.8 V after 3–6 months. Contrast use was not a significant factor in the threshold evolution (*p* = 0.26).

The LVEF increased from 30.2 ± 6.6% preoperatively to 33.4 ± 9.8% within at least two months following the procedure (median follow-up duration 137 days, IQR = 142.5 days). An LVEF increase was observed in all three groups (ΔLVEF = 2.3 ± 8.1% in the NCG, 2.9 ± 7.1% in the COG and 4.1 ± 8.4% in the CG). While the pre- and postoperative values differed (*p* < 0.01), contrast use did not significantly influence the LVEF decrease (*p* = 0.41). Similar results were obtained in the ITT analysis of the QRS, LVL threshold and LVEF evolution. The values are detailed in Table 3.

**Table 3:** Outcome and evolution

	Intention-to-treat			As-treated			
	IG	CG	*p*-value	NCG	COG	CG	*p*-value
LVL Threshold							
Intraoperative Threshold (V)	1.0 ± 0.7	1.1 ± 0.6	0.82	1.0 ± 0.6	1.1 ± 0.7	1.0 ± 0.6	0.18
Postoperative Threshold (V)	1.2 ± 0.8	1.3 ± 1.1	0.29	1.2 ± 0.9	1.2 ± 0.6	1.3 ± 1.1	0.57
Follow-up Threshold (V)	1.1 ± 0.6	1.2 ± 0.9	0.52	1.1 ± 0.5	1.2 ± 0.7	1.2 ± 0.9	0.62
LVL Impedance							
Intraoperative Impedance (Ω)	865.3 ± 299	861.6 ± 641	0.95	851.5 ± 286	889.8 ± 323	861.6 ± 641	0.89
Postoperative Impedance (Ω)	706.8 ± 244	701.7 ± 240	0.85	711.8 ± 244	698.3 ± 246	701.7 ± 240	0.93
Follow-up Impedance (Ω)	786.0 ± 254	781.9 ± 256	0.90	801.3 ± 241	756.7 ± 278	781.9 ± 256	0.66
QRS-Duration							
Preoperative QRSd (ms)	149.8 ± 27.1	157.7 ± 29.1	**0.02**	150.7 ± 26.1	148.2 ± 28.8	157.7 ± 29.1	0.06
Postoperative QRSd (ms)	142.1 ± 23.2	144.0 ± 24.9	0.55	140.3 ± 22.3	145.1 ± 24.6	144.0 ± 24.9	0.47
ΔQRSd (ms)	−7.9 ± 27.3	−13.1 ± 26.3	0.14	−10.4 ± 28.3	−3.9 ± 25.6	−13.1 ± 26.3	0.13
LVEF							
Preoperative LVEF (%)	31.0 ± 7.6	30.8 ± 7.9	0.89	29.9 ± 7.8	33.0 ± 6.6	30.8 ± 7.9	0.59
Follow-up LVEF (%)	33.4 ± 10.8	34.5 ± 7.2	0.56	32.2 ± 10.8	36.2 ± 9.8	32.6 ± 9.9	0.39
ΔLVEF (%)	2.5 ± 7.7	4.1 ± 8.4	0.34	2.3 ± 8.1	2.9 ± 7.1	4.1 ± 8.4	0.62

## Complications

Overall, the perioperative complication rate was 9.5%. The complications included pericardial effusion (3.5%), pneumothorax (2.9%), ICU admission (2.6%), mechanically induced arrhythmia (1.7%) and death (0.9%).

The ITT and AT analyses showed similar results: Although contrast use was associated with CS dissections (5% in the CG, 3.2% in the COG and 0% in the NCG, *p* = 0.03), the postoperative pericardial effusion prevalence, ICU admission rate and mortality rate were not significantly associated with contrast use (*p* > 0.05 for each in the ITT and AT analyses as shown in Table 4). Of the pericardial effusions 8/12 (66.7%) were treated conservatively, while 4/12 required pericardiocentesis. A single death occurred intraoperatively (1/3), while 2/3 occurred postoperatively. Other complications, including intraoperative mechanically induced arrhythmia and pneumothorax, were also not associated with contrast use (*p* > 0.05 in the ITT and AT analyses). Table 4 details the frequencies of the complications.

**Table 4:** Distribution of the complications and revision indication

			Intention-To-Treat			As-Treated				
			IG **(** *n* ** = 167)**	CG **(** *n* ** = 179)**	*p*-value	NCG **(** *n* ** = 105)**	COG **(** *n* ** = 62)**	CG **(** *n* ** = 179)**	*p*-value	Total **(** *n* ** = 346)**
Perioperative Complications			11 (6.6%)	22 (12.3%)	0.07	7 (6.7%)	4 (6.5%)	22 (12.3%)	0.20	33 (9.5%)
	Pericardial effusion (PostOP Echo)		4 (2.4%)	8 (4.5%)	0.23	2 (1.9%)	2 (3.2%)	8 (4.4%)	0.76	12 (3.5%)
		Conservatively treated	3 (1.8%)	5 (2.8%)		2 (1.9%)	1 (1.6%)	5 (2.8%)		8 (2.1%)
		Pericardiocentesis	1 (0.6%)	3 (1.7%)		0 (0.0%)	1 (1.6%)	3 (1.7%)		4 (1.1%)
	Pneumothorax (PostOP Chest Xray)		4 (2.4%)	6 (3.4%)	0.75	2 (1.9%)	2 (3.2%)	6 (3.4%)	0.78	10 (2.9%)
		Conservatively treated	3 (1.8%)	3 (1.7%)		2 (1.9%)	1 (1.6%)	3 (1.7%)		6 (1.7%)
		Chest drain	1 (0.6%)	3 (1.7%)		0 (0.0%)	1 (1.6%)	3 (1.7%)		4 (1.2%)
	IntraOP mechanically induced arrhythmia		1 (0.6%)	5 (2.8%)	0.22	1 (0.9%)	0 (0.0%)	5 (2.8%)	0.33	6 (1.7%)
	Postoperative ICU admission		3 (1.8%)	6 (3.4%)	0.50	2 (1.9%)	1 (1.6%)	6 (3.4%)	0.74	9 (2.6%)
	Death		1 (0.6%)	2 (1.1%)	0.99	1 (0.9%)	0 (0.0%)	2 (1.1%)	0.68	3 (0.9%)
		Intraoperative	0 (0.0%)	1 (0.6%)		0 (0.0%)	0 (0.0%)	1 (0.6%)		1 (0.6%)
		Postoperative	1 (0.6%)	1 (0.6%)		1 (0.9%)	0 (0.0%)	1 (0.6%)		2 (1.1%)
Revision indications			6 (3.6%)	7 (3.9%)	0.88	5 (4.8%)	1 (1.6%)	7 (3.9%)	0.64	13 (3.7%)
	LVL-dislocation or fracture		3 (1.8%)	5 (2.8%)	0.73	2 (1.9%)	1 (1.6%)	5 (2.8%)	0.82	8 (2.3%)
	Phrenic stimulation		1 (0.6%)	1 (0.6%)	0.96	1 (0.9%)	0 (0.0%)	1 (0.6%)	0.73	2 (0.6%)
	Lead-related endocarditis		2 (1.2%)	1 (0.6%)	0.61	2 (1.9%)	0 (0.0%)	1 (0.6%)	0.44	3 (0.9%)

The post-operative GFR alteration was not significantly influenced by the procedure, contrast use or the interaction of both factors (respectively *p* = 0.22, *p* = 0.16 and *p* = 0.86 in the AT analysis; *p* = 0.17, *p* = 0.09 and *p* = 0.72 respectively in the ITT analysis). Regression modelling showed that the baseline creatinine level was a significant predictor of AKI (*p* = 0.02). AKI developed in 12% of the patients in the NCG (baseline creatinine level = 1.4 ± 0.6 mg/dL) and in 7.1% of the patients in the CG (baseline creatinine = 1.2 ± 0.6 mg/dL). Contrast use did not represent a significant effect (*p* = 0.22).

The median device follow-up duration was 2.4 years (IQR = 1.4 years). 13 LVL (3.7%) revisions were performed. The revision rate and indications were not associated with contrast use (*p* = 0.88 in the ITT and *p* = 0.64 in the AT analysis). 8/13 (61.6%) revisions were performed due to LVL dislocation, 3/13 (23%) were completed due to lead-related endocarditis, and 2/13 (15%) were due to phrenic stimulation that could not be resolved through reprogramming. The revision indications were not significantly associated with contrast use (*p* > 0.05).

## Discussion

We present the largest study to date on contrast-free CRT implantation. Our data suggests that an initial contrast-free LVL implant approach was successful in 62.9% of the cases, associated with shorter procedures, less fluoroscopy use, lower radiation doses and similar outcomes and periprocedural complication rates. Avoiding contrast medium use can therefore be more cost-effective by reducing the need for additional materials and shortening the procedure duration without compromising the outcome. In the remaining cases, a crossover to contrast was necessary; however, this was not associated with more adverse events or a longer procedure.

The feasibility of contrast-free LVL implantation in renal insufficiency has been demonstrated^13^. Shorter procedural durations were observed in the contrast-free group compared to the control group, without reaching significance (146 ± 29 vs. 153 ± 25 min, *p* = 0.57, *N* = 17 interventions). Our study furthers this finding, highlighting that this approach can be routinely performed and is associated with significantly shorter interventions (100.8 ± 41 min vs. 131.1 ± 50.0 min, *p* < 0.05).

There is heterogeneity in the reported duration of CRT implants. Our procedure times were consistent with several other groups, such as 102 ± 34 min 22 and 105 ± 54 min 23. Shorter conventional CRT intervention durations as low as 83 min have been recently reported 24 and may reflect exceptional circumstances, although the use of contrast was not explicitly stated. There are many factors that can influence procedural duration, including proceduralist experience, site-specific protocols and the definition of procedure duration used. Thus, to be able to comment on the relative procedural duration of a contrast-free approach, an internal control group is essential.

Comparing contrast-free LVL implantation procedural details with other contrast-sparing techniques: Three-dimensional electroanatomic mapping system (EAMS)-enhanced CRT implantation was associated with higher fully contrast-free success rates (74% 25 vs. 62.9% in our setting). The overall implantation success rates (after inclusion of crossover to contrast use were similar (98% vs. 99%). We note that the reported EAMS enhanced CRT implantations were longer with an average duration of 131 min (median 120 min, range 94155 min) vs. 101 ± 41 min in our setting. This emphasizes the important role that coronary sinus angiography continues to play for CRT implantation, as a backup option in case of failure of lead placement using fluoroscopic landmarks or electrophysiological activation mapping alone.

Posterior, posterolateral and lateral LVL locations are associated with favourable long-term outcomes/LV reverse remodelling in the context of HFrEF with LBBB. Anterior and apical positions are to be avoided. Multiple studies 26, 27 have demonstrated that lateral/posterior wall pacing correlates with greater left ventricular reverse remodelling, improved clinical response and reduced mortality, while anterior or apical positions are associated with suboptimal outcomes and should therefore be avoided. In our study, 96.6% of the LVL could be positioned in a lateral, posterolateral or posterior position without contrast use. While the distribution pattern slightly differs- posterior positions being more frequent without contrast (36.8% vs. 24.3%) and posterolateral more common with contrast (39.2% vs. 35.6%). Both strategies achieved lead positioning predominantly in optimal anatomical locations. In the contrast-free group, 3.4% of the leads were placed anterolaterally, and in the contrast-group we observed only 1 anterolateral case (0.7%) A minority (4.1%) of the leads were placed apically. However, there was no association between the apical positions and contrast use, suggesting there is a subgroup of patients where better lead positioning cannot be achieved regardless of contrast use, for example, due to venous anatomy variations, extensive myocardial scarring or phrenic nerve stimulation.

The intraoperative and short-term follow-up threshold and impedance values aligned with the ESC guideline 28 and were not associated with contrast use, reflecting the similar LVL positions obtained for the studied groups.

We note that longer preoperative QRS durations were observed in the control group, which may reflect a more advanced stage of structural heart failure, which may be associated with more advanced ventricular dilation, myocardial fibrosis and electrical remodelling^29^. In addition, we note that a significantly higher proportion of patients in the CG underwent CRT-upgrade compared to the IG. CRT-Upgrade candidates may present a more advanced heart disease stage, with longer heart failure duration, prior RV Pacing and more pronounced structural and electrical remodelling. These changes may be accompanied by more complex CS anatomy, increasing LVL implantation complexity. In this context, interventionalists may have been more inclined to use CM from the start to facilitate LVL placement.

More complications were observed in the contrast group, with CS dissection being the most prevalent complication (6/17 intraoperative complications), and pericardiocentesis was necessary in 3 cases. The higher prevalence of this complication is likely due to the inherent ability to observe dissections when using contrast. Furthermore, in 5 of the 6 CS observed dissections, an occlusive balloon was used during the venography. This finding is supported by published data showing that occlusive venography is associated with a higher risk of CS trauma compared to direct venography 30, 31. This may be attributed to mechanical trauma caused by the balloon catheter or subselector sheath used for contrast delivery and to barotrauma during balloon inflation. Asymptomatic CS dissections may have occurred in the contrast-free group, but they did not result in a difference in clinical outcome if they did indeed occur. If difficulty in LVL placement in the contrast-free group were due to CS dissection, then this would have resulted in a higher rate of dissections in the crossover group, which we did not observe.

The baseline characteristics of the CG, including longer preoperative QRS duration, higher proportion of CRT upgrades and higher BMIs likely reflect a more advanced stage of heart failure. The presence of more advanced structural disease may inherently be associated with a higher procedural risk. At the same time, it may have necessitated an intensive strategy to ensure optimal LV lead placement, thus influencing the interventionalist’s decision to use contrast from the outset or to rapidly cross over to contrast use.

Procedural workflow may partly explain the observed differences in LV lead locations. CS tributaries were explored sequentially. In the contrast-free group, posterior/posterolateral branches were probed first due to their relative accessibility. If the electrical parameters and mechanical lead stability were satisfactory, the position was accepted. In cases of suboptimal electrical parameters or insufficient mechanical stability, deeper CS intubation was performed or crossover to contrast was considered in case of contrast-free failure. In contrast-guided procedures, venography provided immediate visualization of the venous anatomy, allowing a targeted engagement of the lateral branches.

The use of CM remains relatively contraindicated in patients with documented contrast hypersensitivity or hyperthyroidism, as it can trigger anaphylactic reactions and thyrotoxicosis. For these high-risk groups, alternative contrast-sparing strategies to contrast use or prophylactic measures (when contrast use is unavoidable) are recommended 32, 33. In our study, we did not observe instances of anaphylaxis or thyrotoxicosis. As for renal insufficiency, we observed that AKI occurred in 12% of the patients in the NCG and 7.1% in the CG, that the predictor for renal deterioration was the baseline creatinine level and that contrast use did not represent a significant effect in our study. The AKI incidence is within the range of reported results (7.9% 5, 10.2% 4, 12% 34). The amount of CM used in our study was modest in both the CG (44.1 ± 35.6 mL) and the COG (33.6 ± 22.7 mL), and falls below reported high-risk CM quantity thresholds (> 95 mL) 5. These findings suggest that the use of low CM quantity, if necessary, is not per se a predictor of AKI and that impaired renal function does not strictly mandate a contrast-free approach. While contrast-free implantation represents the prudent initial approach in CKD patients, an early crossover to contrast use should be considered in challenging implantation cases.

Left bundle branch pacing (LBBP) is an emerging alternative technique for achieving left ventricular resynchronisation that inherently does not require contrast use. With a growing body of evidence that LBBP yields equivalent (or superior in non-ischemic heart failure) clinical and echocardiographic outcomes to bi-ventricular pacing 35, 36, it is possible that in the future the cases in our crossover group could be transitioned to left bundle pacing, thus preserving the contrast-free nature of the procedure.

Beyond standard CRT and LBBP, left-bundle branch optimized CRT (LOT-CRT) combines LBBP with an additional LV lead, and has been associated with favourable electrophysiological and hemodynamic outcomes than standalone CRT 37 or LBBP 38 in patients with advanced conduction disease. Our data suggests that LOT-CRT could be achieved without contrast use. This would however require further dedicated research.

### Limitations

This study was performed as a retrospective study and therefore lacks randomization and may be subject to selection bias. We did observe that contrast-free LVL implantation was significantly associated with lower BMIs. In addition to the classical contraindications of contrast use, such as advanced CKD or iodine allergy, the intention to perform the procedure with or without contrast were not randomized may have been influenced by anticipated vein anatomy (for example, extrapolated from previous coronary angiographies). We observed longer preoperative QRS durations and more CRT-upgrade procedures in the control group, which may reflect more pronounced structural and electrical remodelling. This may have influenced the interventionist’s initial strategy and preference to use contrast from the outset. The initial strategy may have also been influenced by interventionist’s individual preference and overall experience with CRT implantation, particularly with contrast-free LVL Implantation. However, analysing the interaction between contrast use and the interventionist as factors for the intervention’s duration showed no interaction between these two factors. To limit the recall bias and bypass eventual documentation inconsistencies, all fluoroscopy films were re-analysed.

Moreover, this study was performed as a single-centre study where CRT implantations are routinely performed, which may limit the generalizability of the results independently of the experience level. Further validation in the context of a prospective randomized controlled multicentre trial is warranted.

## Conclusion

Contrast-free LVL implantation was successfully performed in 2/3 of cases, leading to shorter procedures, less fluoroscopy use, less radiation exposure and fewer intraoperative complications. There was no difference in long-term device performance or complication rate.

Where contrast-free LVL implantation was unsuccessful, an intraprocedural crossover to contrast use did not result in more adverse events or a longer procedure duration than in fully contrast-aided procedures.

For experienced implanters we would suggest that it is reasonable to consider an initial contrast-free approach for CRT implantation.

## Data Availability

No datasets were generated or analysed during the current study.
